# What one patient can teach the next

**DOI:** 10.1002/ctm2.70900

**Published:** 2026-11-14

**Authors:** Ge Zhang, Mingxuan Duan, Tianshu Gu, Qian Guo, Yahui Han, Teng Li

**Affiliations:** ^1^ Department of Cardiology The First Affiliated Hospital of Zhengzhou University Zhengzhou University Zhengzhou China; ^2^ Department of Cardiology Beijing Tsinghua Changgung Hospital School of Clinical Medicine Tsinghua University Beijing China; ^3^ Department of Cardiology Tianjin Institute of Cardiology Tianjin Key Laboratory of Ionic‐Molecular Function of Cardiovascular Disease the Second Hospital of Tianjin Medical University Tianjin China; ^4^ Liverpool Centre for Cardiovascular Science at University of Liverpool Liverpool John Moores University and Liverpool Heart & Chest Hospital Liverpool UK; ^5^ Department of Otorhinolaryngology‐Head and Neck Surgery Zhongnan Hospital of Wuhan University Wuhan China; ^6^ School of Pharmacy Harbin Medical University Harbin China; ^7^ Department of Ophthalmology The First Affiliated Hospital of Zhengzhou University Zhengzhou University Zhengzhou China

1

A treatment developed for one patient may never be tested in a conventional trial. If that patient improves, or suffers serious harm, what should the next clinical team do differently? In our recent Science Letter, we argued that major outcomes from single‐patient trials should be reported publicly and linked to the research that preceded treatment.^1^ But making an outcome visible is only the first step. We must also decide what can reasonably be learned from it.

A recent report brings this problem into focus. Two children with severe KCNT1‐related epilepsy received an experimental antisense oligonucleotide by injection into the spinal canal. Both experienced fewer seizures, but both developed ventricular enlargement or hydrocephalus. When ventricular changes appeared in the second child, knowledge of the first child's complication prompted closer monitoring. Treatment was subsequently paused and, after the protocol was revised, resumed at lower doses.^2^ The first child's experience helped change care even though the cause of the complication was not fully understood. It did not prove that all similar drugs carry the same risk.

That distinction matters. For safety, an earlier case may be relevant because the drug, dose or route of delivery is similar, even when the two patients have different diagnoses. For benefit, the question is harder: would this patient have improved without the treatment? The course of illness before treatment and outcomes meaningful to the patient are essential starting points.^3^ Even a highly accurate prediction of risk cannot answer which treatment will work. A survival model we developed for advanced heart failure, for instance, estimated prognosis rather than the benefit of a particular therapy.^4^ Estimating treatment effects requires a different kind of evidence.^5^

Biological differences make the problem harder still. Patients given the same diagnosis may not have the same underlying disease state. In our study of atherosclerosis, single‐cell and bulk transcriptomic analyses identified distinct molecular subtypes related to smooth muscle cell states.^6^ These findings suggest questions for further study, not established differences in treatment response. If a patient fails to improve, did the drug fail to affect its intended target? Or did it reach the target without restoring function? Those explanations point to different measurements and experiments.

AI could help clinicians and researchers make these distinctions (Figure 1). Instead of labelling two patients simply ‘similar’, it could retrieve earlier cases relevant to a specific question and show why they matter. A possible safety signal calls for details of exposure and event timing; an apparent benefit calls for baseline disease course and reliable outcome measures. Where molecular findings suggest competing explanations, computational models could help select an experiment to test them.^7^ None of these steps replaces clinical judgment or turns an association into proof of treatment benefit.

**FIGURE 1:**
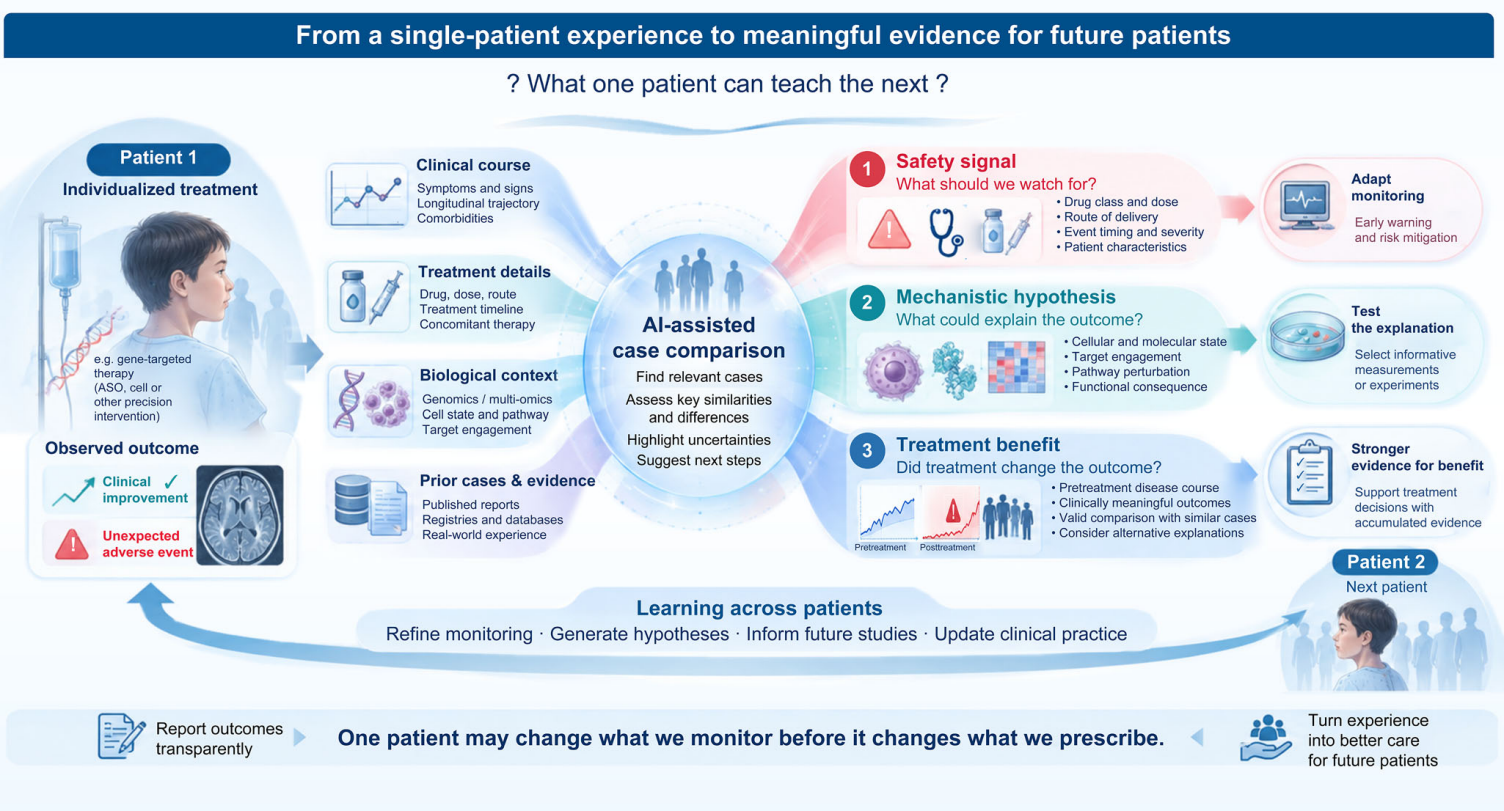
What one patient can teach the next. Timely reporting of outcomes from individualised treatment provides a foundation for learning across patients. Artificial intelligence (AI)‐assisted comparison of clinical trajectories, treatment exposure, biological context, and prior evidence may identify relevant similarities, differences, and uncertainties. These insights can inform three distinct but complementary actions: adapting safety monitoring, testing mechanistic hypotheses, and evaluating treatment benefit against appropriate comparative evidence. Importantly, observations that guide monitoring or further investigation do not necessarily establish treatment efficacy or causality.

This gives case sharing a practical purpose. Existing collaborations for individualised therapies already collect experience across patients and research groups.^8^ An account of an earlier case should make clear what was observed, what remains uncertain and what another team might reasonably change: monitoring, further investigation or, only with stronger evidence, treatment. Major outcomes should be reported promptly without waiting for a complete mechanistic explanation, while detailed patient‐level data remain subject to consent and privacy safeguards. The next patient may benefit first from better monitoring. A treatment decision demands more.

## CONFLICT OF INTEREST STATEMENT

The authors declare no conflict of interest.
